# Recent advances in our understanding of the structure and function of more unusual cation channels

**DOI:** 10.12688/f1000research.17163.1

**Published:** 2019-01-30

**Authors:** Brandon M. Brown, Hai M. Nguyen, Heike Wulff

**Affiliations:** 1 Department of Pharmacology, School of Medicine, University of California, Davis, USA

**Keywords:** cation channel

## Abstract

As their name implies, cation channels allow the regulated flow of cations such as sodium, potassium, calcium, and magnesium across cellular and intracellular membranes. Cation channels have long been known for their fundamental roles in controlling membrane potential and excitability in neurons and muscle. In this review, we provide an update on the recent advances in our understanding of the structure–function relationship and the physiological and pathophysiological role of cation channels. The most exciting developments in the last two years, in our opinion, have been the insights that cryoelectron microscopy has provided into the inner life and the gating of not only voltage-gated channels but also mechanosensitive and calcium- or sodium-activated channels. The mechanosensitive Piezo channels especially have delighted the field not only with a fascinating new type of structure but with important roles in blood pressure regulation and lung function.

## Introduction

Cation channels mediate the flow of cations (Na ^+^, K ^+^, Ca ^2+^, and Mg ^2+^) across hydrophobic lipid membrane barriers, allowing both excitable cells such as neurons or muscle and non-excitable cells such as lymphocytes and endothelial cells to regulate membrane potential, Ca ^2+^ signaling, and various other cellular processes ^1^. Based on the International Union of Basic and Clinical Pharmacology (IUPHAR) *Guide to Pharmacology* ^2^, ** the human genome contains 145 voltage-gated-like channels ^3^, 55 ligand-gated channels ^4^, and 27 “so-called” other channels ^5^, such as connexins or Piezo and store-operated channels, that conduct cations. All of these channels play specific physiological roles, which can make them attractive drug targets for the treatment of disease. Conversely, mutations in cation channel genes or genes encoding associated β-subunits or scaffolding proteins can cause so-called channelopathies such as cystic fibrosis, long or short QT syndrome, and various forms of epilepsy. In this review, we provide an update on what, in our opinion, constitutes the most exciting new findings concerning cation channels during the last two years, namely our increased understanding of gating mechanisms of more unusual, non-voltage-gated channels such as Slack (K _Na_1.1), K _Ca_3.1 (SK4), ENaC, TCP1, or Piezo1. We would like to apologize upfront to the many scientists whose fascinating work we cannot mention due to space constraints or because, as one of our colleagues once so fittingly put it, “we play in a different sand box” and are biased by what we find interesting.

## Advances in understanding cation channel structures

The recent increase in the number of solved ion channel structures is largely due to technological advances in cryoelectron microscopy (cryo-EM), such as improvements in microscope design and imaging hardware, as well as enhanced image processing which allows the reconstruction of 3D structures from a large number of single-particle 2D images even if they are conformationally heterogeneous ^6^. Cryo-EM is thus increasingly being used to obtain near-atomic resolution structures ^6^ of membrane proteins such as G protein–coupled receptors (GPCRs) and ion channels that have traditionally been difficult to obtain with x-ray crystallography or nuclear magnetic resonance spectroscopy. (See Table 1 for the Protein Data Base ID numbers of the structures mentioned in this article.) Overall, the most impressive effort in channel structure–function studies within the last couple of years has come from the laboratory of Roderick MacKinnon, whose group solved the first ion channel structure, the bacterial K ^+^ channel KcsA, in 1998 ^7^. Since the latter part of 2016, his group has elucidated the structure of Eag1 (K _v_10.1) ^8^, which unlike previously solved K _V_ channel structures was not domain-swapped; hERG (K _V_11.1) ^9^; KCNQ1 (K _V_7.1) ^10^; HCN1 ^11^; Slack (Slo2.2, K _Na_1.1) ^12^; Kir6.2 in complex with SUR1 ^13^; BK (K _Ca_1.1) in the presence and absence of calcium ^14, 15^; as well as the human K _Ca_3.1 (SK4) channel in complex with calmodulin ^16^. All of these structures provided new and often unexpected insights into the gating mechanisms of the respective channels and showcase the power of cryo-EM.

**Table 1.** Ion Channel Structures.

Protein Data Base ID	Channel	Species	Method	Resolution	Conformation
6CNM	K _Ca_3.1 (SK4, IK)	Human	Cryo-EM	3.4 Å	Closed state
6CNN	K _Ca_3.1 (SK4, IK)	Human	Cryo-EM	3.5 Å	Ca ^2+^-bound open state I
6CNO	K _Ca_3.1 (SK4, IK)	Human	Cryo-EM	4.7 Å	Ca ^2+^-bound open state II
5VA1	K _V_11.1/hERG	Human	Cryo-EM	3.7 Å	Open state
5VA2	K _V_11.1/hERG	Human	Cryo-EM	3.8 Å	Open state
5VA3	Kv11.1/hERG	Human	Cryo-EM	4.0 Å	Open state
5U70	K _Na_1.1 (Slack, Slo2.2)	Chicken	Cryo-EM	3.8 Å	Open state
5U76	K _Na_1.1 (Slack, Slo2.2)	Chicken	Cryo-EM	3.8 Å	Closed state
6BQN	ENaC	Human	Cryo-EM	3.9 Å	Uncleaved state
6C96	TPC1	Mouse	Cryo-EM	3.4 Å	Closed state
6C9A	TPC1	Mouse	Cryo-EM	3.2 Å	Open state
5Z10	Piezo1	Mouse	Cryo-EM	4.0 Å	Closed state
6B3R	Piezo1	Mouse	Cryo-EM	3.7 Å	Closed state
6BPZ	Piezo1	Mouse	Cryo-EM	3.8 Å	Closed state
5K7L	K _V_10.1 (Eag1)	Rat	Cryo-EM	3.8 Å	CaM-bound state
5TJ6	K _Ca_1.1 (Slo1, BK)	Aplysia	Cryo-EM	3.5 Å	Open state
5TJI	K _Ca_1.1 (Slo1, BK)	Aplysia	Cryo-EM	3.8 Å	Ca ^2+^-free state
5U6O	HCN1	Human	Cryo-EM	3.5 Å	Human HCN1 in cAMP-free closed state
5U6P	HCN1	Human	Cryo-EM	3.5 Å	Human HCN1 in cAMP-bound open state
6C3O	K _ATP_ (K _IR_6.2-SUR1)	Human	Cryo-EM	3.9 Å	Quatrefoil form
6C3P	K _ATP_ (K _IR_6.2-SUR1)	Human	Cryo-EM	5.6 Å	Propeller form
6DVW	TRPV3	Mouse	Cryo-EM	4.3 Å	Closed apo state
6DVY	TRPV3	Mouse	Cryo-EM	4.0 Å	2-APB–bound closed state
6DVY	TRPV3	Mouse	Cryo-EM	4.2 Å	2-APB–bound open state
6MHO	TRPV3	Human	Cryo-EM	3.4 Å	Closed apo state
6MHS	TRPV3	Human	Cryo-EM	3.2 Å	Sensitized state
6BPQ	TRPM8	Collared flycatcher	Cryo-EM	4.1 Å	
6DJR	TRPC3	Human	Cryo-EM	5.8 Å	Apo state
6DJS	TRPC3	Human	Cryo-EM	5.8 Å	Apo state
6A70	PKD1/PKD2	Human	Cryo-EM	3.6 Å	Closed state
5XSY	Na _V_1.4-β1	Electric eel	Cryo-EM	4.0 Å	VSDs in “up” conformation
6AGF	Na _V_1.4-β1	Human	Cryo-EM	3.2 Å	VSDs in “up” conformation
5X0M	Na _V_PaS	Cockroach	Cryo-EM	3.8 Å	Closed pore
6A90	Na _V_PaS	Cockroach	Cryo-EM	2.8 Å	With Dc1a
6A95	Na _V_PaS	Cockroach	Cryo-EM	2.6 Å	With tetrodotoxin and Dc1a
6A91	Na _V_PaS	Cockroach	Cryo-EM	3.2 Å	With saxitoxin and Dc1a
IK4C	KcsA	*Escherichia coli*	X-ray	2.0 Å	Closed, ready to open
IK4D	KcsA	*E. coli*	X-ray	2.3 Å	Closed, inactivated state
5VK6	KcsA(E71A)	*E. coli*	X-ray	2.3 Å	Open conductive state
5VKE	KcsA(Y82A)	*E. coli*	X-ray	2.4 Å	C-type inactivated state
5GJV	Ca _V_1.1	Rabbit	Cryo-EM	3.6 Å	Inactivated state
6C1P	Na _V_Ab(R2G)	*Arcobacter butzleri*	X-ray	2.9 Å	VSD mutation inducing a pathogenic gating pore
6C1M	Na _V_Ab(R2G)	*A. butzleri*	X-ray	2.5 Å	With methylguanidinum
6C1K	Na _V_Ab(R2G)	*A. butzleri*	X-ray	2.7 Å	With guanidnium
6C1E	Na _V_Ab(R3G)	*A. butzleri*	X-ray	2.9 Å	VSD mutation inducing a pathogenic gating pore

2-APB, 2-aminoethoxy-diphenyl borate; CaM, calmodulin; cAMP, cyclic adenosine monophosphate; cryo-EM, cryoelectron microscopy; VSD, voltage sensor domain.

In the most recently solved structure of the intermediated-conductance calcium-activated potassium channel K _Ca_3.1 (SK4), the channel-CaM complex is captured at 3.4- and 3.5-Å resolution in what is suggested to be the closed and two different, activated states. K _Ca_3.1 is widely expressed in the immune system, whereas the closely related small-conductance K _Ca_2 (SK1-3) channels are found mostly in neurons. The K _Ca_3.1 structure shows that the channel is non-domain-swapped ^16^. In the absence of calcium, calmodulin is bound to the calmodulin-binding domain (CaM-BD) in the C-terminus with its C-lobe while the N-lobe is poorly resolved. However, the true game changer for our understanding of K _Ca_ channel gating—and possibly its drug development efforts—are the two open structures, where the N-lobe of calmodulin has “swung over” to the S4-S5 linker of another subunit and pulls it down, causing S6 to move outward and to expand the cytoplasmic pore entry. The structure thus solves the long-standing problem of the gating symmetry and shows that K _Ca_ channels in fact gate with fourfold symmetry and not with twofold symmetry as previously suggested ^17^. A very interesting technical aspect of this article is how the heterogenicity of the particles with the “floppy” N-lobe in the absence of calcium was dealt with and how the two different open states were sorted out. We therefore highly recommend the online methods of the article ^16^ to interested readers. Another powerful illustration of the advances of cryo-EM is the Hite *et al*. study ^18^, in which the MacKinnon group captured the sodium-activated potassium channel Slack (K _Na_1.1, Slo2.2) in multiple conformations by titrating increasing concentrations of sodium. Slack is expressed in neurons, where it is activated by high concentrations of intracellular Na ^+^ that can occur following repeated action potential firing. Together with the previously solved closed Slack structure ^12^, the Hite and MacKinnon article, which collected images at five different Na ^+^ concentrations, shows that Slack exists in multiple closed conformations from which an open conformation emerges in a highly Na ^+^-dependent manner ^12^, suggesting that opening of this ligand-gated channel is a highly concerted, switch-like process.

In another effort from the MacKinnon laboratory, the structure of the hyperpolarization-activated cyclic nucleotide-gated (HCN) channel member 1 was elucidated ^11^. HCN channels are non-selective cation channels that are permeant to both sodium and potassium and underlie pace making both in the heart and in the central nervous system. All four members of this family are voltage-gated but also are modulated by the endogenous ligand cyclic adenosine monophosphate (cAMP). The structure, which was solved at 3.5-Å resolution in the presence and absence of cAMP, explains the 4:1 potassium-to-sodium permeability ratio of HCN and shows an unusually long S4 helix that extends in the cytoplasm to stabilize a closed pore in the presence of a depolarized voltage sensor. These structural features suggest a gating mechanism in which downward displacement of the S4 helix during membrane hyperpolarization disrupts these stabilizing interactions to open the channel, thus explaining HCN’s reversed polarity of voltage dependence. Binding of cAMP induces a rotation of the gate-forming inner helices, thus favoring channel opening ^11^.

Transient receptor potential (TRP) channels act as sensors in a variety of physiological processes. They are a diverse group of cation channels that are often relatively non-selective and permeable to sodium, calcium, and magnesium. Recently, a series of TRP channel structures have been solved. The full-length mouse vanilloid subfamily member 3 (TRPV3) was obtained in both the closed state and in the open state with the agonist 2-aminoethoxy-diphenyl borate (2-APB) ^19^. 2-APB was found to bind at three allosteric sites, and channel opening was shown to induce conformational changes in both the outer pore and the intracellular gate ^19^. Understanding TRPV3 activation at the molecular level is important because the channel constitutes a potential target for the treatment of inflammatory skin conditions, itch, and pain. TRP melastatin cation channel member 8 (TRPM8) is the primary cold and menthol sensor in humans. Its structure was recently solved by Yin *et al*. at 4.1-Å resolution ^20^. Whereas the channel shares the same overall homotetrameric structure that is characteristic of other TRP channels, TRPM8 has a unique N-terminal domain fold pattern. The structure shows that the menthol-binding site is located within the voltage sensor–like domain ^20^. Another recently elucidated TRP channel is TRPC3, the TRP cation channel subfamily C member 3, a calcium-permeant channel in which genetic mutations have been associated with neurodegenerative and cardiovascular diseases. The cryo-EM structures for the full-length human TRPC3 and its cytoplasmic domain (CPD) showed that the TRPC3 transmembrane domain resembles other TRP channels and that the CPD is a stable module involved in channel assembly and gating ^21^. The structure of TRPC3 is important for understanding TRP channel gating since it was observed that horizontal helices in the cytoplasmic domain transition into a coiled-coil bundle. These helices are coupled to the transmembrane domain via loops and these transitions facilitate gating. Completing the recent insights into TRP channel structures is the 3.6-Å resolution structure of a truncated human polycystic kidney disease (PKD)1-PKD2 complex in a 1:3 PKD1-to-PKD2 stoichiometry ^22^. Mutations in PKD1 and PKD2 account for most cases of autosomal dominant PKD, rendering this structure very important because of the potential insights it can provide into PKD disease mechanisms by finally affording the opportunity to map a large number of disease mutations onto a structure. PKD1 exhibits a typical voltage-gated ion channel fold that interacts with PKD2 to form a domain-swapped, non-canonical TRP channel. However, many questions remain about this mysterious complex. For example, the S6 segment of PKD1 is broken in the middle, and the extracellular half, S6a, resembles a typical pore helix, whereas the intracellular parts are disordered. The structure further shows three positively charged residues protruding into the putative ion-conducting path, suggesting that the structure is of non-conductive state ^22^.

The membrane protein, Piezo1, encoded by the piezo gene is a 38-transmembrane domain mechanosensitive ion channel that functions as a trimer. Piezo1 is expressed in blood vessels and is crucial for sensing blood flow–associated shear stress. Following up on an earlier, lower-resolution structure ^23^ that showed a trimeric propeller-like protein with a central cap suggesting that Piezo1 uses its peripheral blades as force sensors to gate the central ion-conducting pore, Zhao *et al.* recently solved Piezo1 with an overall resolution of 3.97 Å based on the analysis of close to three million particles ^24^. The new structure shows a central, 90 Å–long intracellular beam, which undergoes a lever-like motion to connect a set of transmembrane helical units to the pore. This structure provides the basis for mechanical activation of Piezo1, in which a lever-like mechanogating mechanism involving the curved blades of Piezo transmits a conformational change to the pore that allows ion permeation. The overall structure and the suggested gating mechanism are unlike those of any other ion channel. The unique features of Piezo1 add breadth and depth to the ways in which ion channels gate and conduct ions.

Another recently solved cryo-EM structure is that of the epithelial sodium channel (ENaC), which regulates Na ^+^ and water homeostasis ^25^. Whereas most sodium channels are voltage-gated and generally consist of a large α subunit with four repeat domains, ENaC assembles as a heterotrimeric channel that contains protease-sensitive domains critical for gating. The structure revealed that ENaC assembles with a 1:1:1 stoichiometry of α:β:γ subunits arranged in a counter-clockwise fashion with the protease-sensitive inhibitory domains wedged between the subunits. Solving the structure of ENaC has allowed for the exact definition of subunit arrangement and stoichiometry and for the elucidation of the mechanism of inhibition. The sodium channel field has been further enriched by two new Na _V_ channel structures, both of which could be very useful for drug design: (1) the first human Na _V_ channel in the Na _V_1.4-beta1 complex at 3.2-Å resolution ^26^ and (2) the insect NaVPaS channel bound to a gating modifier toxin at 2.8-Å resolution and in the presence of tetrodotoxin at 2.6 Å ^27^.

Organellar two-pore channels (TPCs) are an interesting type of channel that, as the name suggests, has two pores. Recently, the structure of the murine sodium-selective TPC1, a channel expressed on endosomes and fulfilling a critical role in regulating the physiological functions of these acidic organelles, was solved ^28^. Not to be confused with the two-pore potassium-selective channels (K _2P_), TPC1 is a homodimer consisting of two six-transmembrane (6-TM) subunits with the N- and C-termini located on the cytoplasmic side of the membrane. The channel is voltage-dependent but can also be activated by phosphatidylinositol 3,5-bisphosphate binding in one 6-TM domain with the other 6-TM domain sensing voltage ^28^. This mechanism of activation is very important since it has recently become clear that phospholipids play an integral role in the activation and regulation of some ion channels.

Lastly, Cuello *et al.* significantly contributed to the field’s understanding of gating by providing a series of structural snapshots showing the gating cycle of KcsA by using traditional x-ray crystallography and a set of cleverly cross-linked constitutively open-channel mutants that capture KcsA in the closed inactivated state and an open-conductive and a deep C-type inactivated state ^29^. These roughly 2-Å structures of KcsA provide unprecedented insight into how the selectivity filter backbone changes as a channel progresses through its gating cycle ^29^.

## Advances in understanding cation channel physiology and pathophysiology

As mentioned in the Introduction, cation channels are best known for their control of membrane potential ^1^ and were traditionally classified into voltage-gated ^3^ and ligand-gated ^4^. However, research in the past decade has started to shed light on the physiological role of channels gated by other mechanisms such as mechanical force ^30– 32^, and we recently have learned about the important roles of Piezo channels in blood pressure regulation and breathing. Furthermore, the field is gaining more and more insights in the role of “silent” channel subunits and cation channels in mitochondria.

Mechanotransduction is a crucial physiological process that is evolutionarily conserved across many species of vertebrate and invertebrate animals down to plants and single-cell protozoans. Despite their long-recognized role in the conversion of mechanical stimuli into biological signals that drive many physiological processes, including itch and pain sensation in vertebrate mammals ^33, 34^, root formation in plants ^35^, and direction changes in ciliates ^36^, it was not until 2010 that the first mechanosensitive cation channels, Piezo1 and Piezo2, were identified ^37^. Since their discovery, the Piezos have not only provided us with fascinating channel structures and channel-gating mechanisms ^23, 24^ but also helped advance our understanding of many physiological processes. Blood pressure regulation is one such process in which mechanosensitive channels are important. Physical activities such as exercise require increased delivery of oxygen-rich blood to the body as oxygen consumption is increased. Vasoconstriction can elevate blood pressure to maximize blood distribution, but it is not clear how changes in blood flow elicit this vascular response. Using a conditional gene knockout (KO) model of Piezo1 ^38^, which is abundantly expressed in the vascular endothelium, Rode *et al*. identified this mechanosensitive channel as the missing link between blood flow and vasoconstriction ^38^. Flow-associated mechanical force changes activate Piezo1 to depolarize the endothelium and the adjacent vascular smooth muscle cells, leading to vasoconstriction induced by the calcium increase via voltage-gated Ca ^2+^ channels ^38^.

Atherosclerosis is a risk factor in stroke and myocardial infarction and occurs selectively in regions where blood flow in the arterial system is disturbed. Interestingly, Piezo1 is also involved in enabling endothelial cells to distinguish different flow patterns ^39^. Both laminar and disturbed flow activate a common regulatory cascade consisting of the mechanosensitive Piezo1 channel, the purinergic P2Y7 receptor, and the Gq/G11-mediated signaling pathway. It turns out, however, that only disturbed flow results in Piezo1- and Gq/G11-mediated integrin activation. Subsequently, the resulting downstream activation of the nuclear factor-kappa B (NF-κB) transcription factor is a key factor underlying the flow-specific regulation of endothelial inflammation in vascular disorders such as atherosclerosis ^39^. Mechanotransduction also plays a crucial role in the respiratory system, in which dysfunction can cause perinatal mortality and adult sleep apnea. Piezo2 is expressed in airway sensory neurons and recently was revealed to be the main sensor behind the transduction of stretch signals associated with breathing. Nonomura *et al.* demonstrated that gene silencing of the Piezo2 channel, both globally and conditionally in airway sensory neurons, led to respiratory distress and mortality ^40^. Conversely, opto-activation of Piezo2 in vagal neurons causes apnea in adult mice, suggesting that Piezo2 is the stretch sensor responsible for mechanotransduction within various airway-innervating sensory neurons ^40^.

Ion channel research is often assumed to be synonymous with patch-clamp electrophysiology because much of our understanding of ion channels comes from studies measuring current flowing through channels. Thus, non-conducting or “silent” channel subunits are often ignored. In most cases, silent channels share high structural and sequence homology with their canonical ion-conducting partners and, when interacting within a macromolecular complex, can fine-tune the biophysical gating properties of their binding partners. The K _V_8.2 channel is one such non-conducting channel that has recently been shown to be not so “silent”. Human carriers of K _V_8.2 mutations suffer severe visual impairment, and the underlying channelopathology was unmasked to involve altered interactions between K _V_8.2 and the K _V_2.1 channel that regulates the excitability of rods and cones ^41^.

The ion channel field has also recently been looking deep below the plasma membrane where cation channels are increasingly being recognized for their importance in intracellular organelles. One such intracellular location, where ion channels are functionally relevant, is the metabolic powerhouse of the cell, the mitochondria. It was of course known for a long time that mitochondrial K ^+^ (mitoK) channels exist and can exert cardioprotective functions, even though their exact molecular identity was unclear. K _Na_1.2 (Slick, Slo2.1) is one of the mitoK channels that had been linked to a cardioprotective role but its expression in mitochondria had never been functionally confirmed by electrophysiology. Through single-channel patch clamp on mitoplasts prepared from cardiomyocytes, Smith *et al*. identified six channels with biophysical and pharmacological properties matching the biophysical characteristic of K _Na_1.2 in wild-type (WT) but not in K _Na_1.2-KO mice ^42^. The authors further demonstrated that the K _Na_ opener bithionol uncoupled respiration in WT but not KO cardiomyocytes and that KO mice had elevated body fat and that their hearts were less responsive to increases in energy demand, thus confirming a role of K _Na_1.2 in the regulation of energy consumption and fat metabolism ^42^.

Last, but certainly not least, the cation field recently was afforded an atomic-level view into the pathogenic mechanisms of periodic paralysis, a channelopathy with episodes of flaccid muscle weakness, which is caused by point mutations in the S4 segment of the voltage sensor domain of either the voltage-gated sodium channel Na _V_1.4 or the voltage-gated calcium channel Ca _V_1.1. These mutations, which affect the arginine residues (R1, R2, or R3) in S4 that act as gating charges, create a “leaky” voltage sensor through which cations can permeate. By introducing these mutations into the bacterial Na _V_Ab channel and solving its structure at 2.7-Å resolution by x-ray crystallography, the Catterall laboratory ^43^ visualized the pathway for this cation leak and identified a possible binding site for the design of drugs to possibly treat hypokalemic and normokalemic periodic paralysis.

## Conclusions

With the recent advances in cryo-EM techniques, the cation channel field has gained a new understanding of ion channel structure–function relationships with never-seen-before molecular details during the last two years. Although it has often been pointed out that the conditions under which cryo-EM is performed are very unphysiological, we believe that the field is getting closer to understanding the full gating cycle of both voltage- and ligand-gated channels at the atomistic level as they progress from the resting to the open and the inactivated state. Of course, ion channel function is inherently dynamic, and a complete understanding of the mechanisms of gating ^44^ and permeation and selectivity ^45^ as well as the visualization of ion flow at the atomistic level ^44^ will only be possible with the aid of molecular dynamics (MD) simulations.

Whether these structures will truly enable structure-based drug design and accelerate ion channel drug discovery is a different question in our opinion. Drug development has many aspects: first and foremost, target validation, which is why it is crucial to gain what is often termed “deep” understanding of the biology of ion channels. As the last two years have again demonstrated, we are still learning new biology, as revealed by the unexpected role of the Piezo channels in blood pressure regulation and breathing or the importance of mitochondrial K _Na_1.2 channels in energy consumption and fat metabolism.
